# Sex-Specific *Wolbachia* Infection Patterns in Populations of *Polygraphus proximus* Blandford (Coleoptera; Curculionidae: Scolytinae)

**DOI:** 10.3390/insects11080547

**Published:** 2020-08-18

**Authors:** Roman Bykov, Ivan Kerchev, Marya Demenkova, Artem Ryabinin, Yury Ilinsky

**Affiliations:** 1 Laboratory of Molecular Genetics of Insects, Institute of Cytology and Genetics SB RAS, 630090 Novosibirsk, Russia; bykovra@bionet.nsc.ru (B.R.); judina@bionet.nsc.ru (D.M.); art@bionet.nsc.ru (R.A.); 2 Laboratory of Insect Pathology, Institute of Systematics and Ecology of Animals SB RAS, 630091 Novosibirsk, Russia; ivankerchev@gmail.com; 3 Institute of Living Systems, Immanuel Kant Baltic Federal University, 236041 Kaliningrad, Russia

**Keywords:** *Polygraphus proximus*, Scolytinae, *Wolbachia*, invasive range, native range, infection rate, *Wolbachia* titer

## Abstract

**Simple Summary:**

*Wolbachia* bacteria are the most common symbionts of insects. These bacteria are ordinarily transmitted via oocyte cytoplasm from mother to progeny, like mitochondria, and are sporadically transmitted from one species to another. The *Wolbachia* symbionts have evolved to be parasitic (feminization of genetic males, male-killing, parthenogenesis, and cytoplasmic incompatibility) or/and mutualistic (increasing lifespan and fecundity, providing vitamins and nutrients, defending against viruses and parasites). Here we have studied *Wolbachia* infection in populations of four-eyed fir bark beetle *Polygraphus* *proximus*, which is one of the most dangerous pests of Siberian fir forests. A high rate of the only *w*Prox *Wolbachia* strain in *P.* *proximus* populations was found in a vastly studied territory. Surprisingly, females were more often harboring *Wolbachia* than males. Besides, a comparison of the *Wolbachia* density in individuals has revealed that females contain much more *Wolbachia* symbionts than males. We suppose that the difference in infection status, as well as the difference in *Wolbachia* load between males and females within a population, can be found in some other *Wolbachia*–host associations.

**Abstract:**

*Wolbachia* symbionts are maternally inherited bacteria that are widely distributed among Arthropoda hosts. *Wolbachia* influence their host biology in diverse ways. They may induce reproductive abnormalities, protect hosts against pathogens and parasites, or benefit hosts through metabolic provisioning. The progeny of an infected female are ordinarily infected with *Wolbachia*; however, *Wolbachia* have no future in male host progeny because they cannot transmit the symbiont to the next generation. Here, we analyze native and invasive populations of the four-eyed fir bark beetle (*Polygraphus proximus*) for *Wolbachia* prevalence and symbiont genetic diversity. This species is a dangerous pest of Siberian fir (*Abies sibirica*) forests. The native range of *P. proximus* includes the territories of the Russian Far East, Japan, Korea, and Northeast China, whereas its invasive range includes West Siberia, with further expansion westward. Surprisingly, we revealed a difference in the patterns of *Wolbachia* prevalence for males and females. Infection rate and *Wolbachia* titers were higher in females than in males. ST-533, the only haplotype of *Wolbachia* supergroup B, was associated with a minimum of three out of the five described mitochondrial haplotypes.

## 1. Introduction

Bacteria of the *Wolbachia* genus are the most successfully inherited symbionts that inhabit numerous arthropod and nematode hosts [1,2,3]. *Wolbachia* are vertically transmitted through host generations via oocytes [4], and are sporadically transmitted, horizontally, to non-related hosts [5,6,7,8,9]. These symbionts may affect their host biology in different ways, from manipulating host reproduction to increasing host fitness. Thus, *Wolbachia* increase lifespan and fecundity [10,11,12], protect against viruses and parasites [13,14,15,16], provide vitamins and other nutrients [17,18], and suppress some mutations in their hosts [19,20,21]. They also influence host reproduction by inducing feminization of genetic males, male killing, parthenogenesis, or cytoplasmic incompatibility (CI) [22]. These phenomena promote the spread of infection in a host population. The first three phenomena lead to a shift in the sex ratio towards females in a host population [22], whereas CI is manifested by embryonic mortality in the progeny of symbiont-free females and *Wolbachia*-harboring males. Therefore, *Wolbachia* in males commonly have no future and, in the case of CI, only function in controlling the spread of the symbiont in a host population.

**Figure 1:**
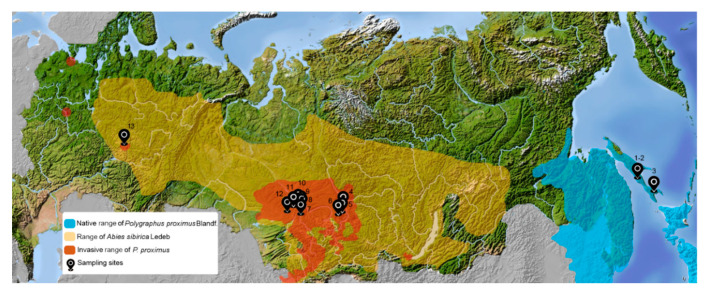
Map of the native and invasive ranges of *Polygraphus proximus* and the range of *Abies sibirica*. Sampling sites for *P. proximus* are indicated (the same numbers in Table 1 and Table 2): 1—Tomarinsky-1; 2—Tomarinsky-2; 3—Yuzhno-Sakhalinsk (Sakhalin province); 4—Bolshemurtinsky district; 5—Emelianovsk forestry; 6—Kozulsk forestry (Krasnoyarsk krai); 7—Tomsky; 8—Krivosheinsky; 9—Molchanovsky; 10—Verkhneketsky; 11—Chainsky; 12—Bakcharsky (Tomsk province); 13—Malopurginsky district (Udmurtia).

We studied *Wolbachia* infection in native and invasive populations of the four-eyed fir bark beetle *Polygraphus proximus* Blandford, 1894 (Coleoptera; Curculionidae: Scolytinae). This species is one of the most dangerous pests of Siberian fir (*Abies sibirica*) forests. Since 2014, *P. proximus* has been listed in the European and Mediterranean Plant Protection Organization (EPPO) Alert List (https://gd.eppo.int/taxon/POLGPR). The native range of *P. proximus* includes the territories of the Russian Far East, Japan, Korea, and Northeast China [23,24,25]. The secondary (invasive) range covers the territories of West Siberia [24,25], with further expansion occurring westward (Figure 1).

*Wolbachia* infections have been reported in Scolytinae species, particularly in the following genera: *Coccotrypes*, *Euwallacea*, *Hypothenemus*, *Ips*, *Pityogenes*, *Taphrorychus*, *Xyleborus*, and *Xylosandrus* [26,27,28,29,30]. The infection rates vary, but are above 70% in most cases. The majority of *Wolbachia* isolates belong to supergroup A, with a few belonging to supergroup B.

Here, we first report on *Wolbachia* in the four-eyed fir bark beetle and determine infection rates of the population, including the infection prevalence according to sex. To do so, we carried out *Wolbachia* titer by qPCR and genotyped *Wolbachia* isolates using a multilocus sequence typing (MLST) protocol [31]. In addition, the barcode region of mitochondrial DNA was sequenced for infected and uninfected individuals collected from different localities, because *Wolbachia* symbionts and mitochondria are inherited together, according to the host’s maternal lineage, and can therefore form cytotypes. A cytotype results from the mitotype and the genotype of an infection, meaning cytotypic analysis can reveal the evolutionary history of *Wolbachia* infection [6,32,33,34,35].

## 2. Materials and Methods

### 2.1. Insect Collection

Adult specimens of *P. proximus* were collected in four regions of Russia: Sakhalin province (native range) (N = 60), Tomsk province (N = 271), Udmurtia (N = 38), and Krasnoyarsk Krai (N = 48) (invasive territories) (Figure 1 and Table 1). In Tomsk province and Udmurtia, the beetles were collected in their parental nests, whereas in Krasnoyarsk they were collected from forest products. In Sakhalin, overwintered beetles and those intruding into the bark of a host tree were collected. The samples were stored in air-dry conditions or fixed in 96% ethanol. The primary study was designed to analyze the infection status of each individual regardless of beetle sex. However, in the course of the study, we had to change the design to specifically include sexes as defined according to forehead morphology [36]. Finally, 7 out of 13 samples were subdivided by sex, namely one from Sakhalin province (beetles forming pairs), one from Udmurtia, two from Tomsk province, and three from Krasnoyarsk krai.

### 2.2. DNA Extraction

The total DNA was individually extracted from whole specimens. The insects were homogenized in 200 µL of DB extraction buffer (10 mM Tris-HCl (pH 8.0), 25 mM EDTA, 0.5% SDS, 0.1 M NaCl) with 1 µL of proteinase K (AppliChem) and incubated at 56 °C for 1 h. Total DNA was purified with NaOAc, precipitated, and dissolved in 100 µL of double-distilled water.

### 2.3. PCR and Sequencing

All DNA samples were tested by PCR with HCO/LCO primers for the cytochrome c oxidase subunit I (*COI*) gene [37]. *Wolbachia* infection status was determined by conventional PCR using the W-SpecF/R primer set [38] and coxAf1/r1 [31], and additionally confirmed for random samples with primer sets hcpAf1/r1 and fbpAf1/r1 [31]. *Wolbachia*-negative samples and controversial samples were checked by nested PCR with ftsZunif [39] for the first round and ftsZf1/r1 for the second, and/or fbpAf2/r2 and fbpAf1/r1, respectively. The PCR reactions were performed using BioMaster HS-Taq PCR (2×) (BiolabMix, Novosibirsk, Russia) with a 20 µL volume. The thermocycler protocol was 95 °C for 5 min, followed by 35 cycles of conventional PCR, and 15 + 30 cycles of nested PCR at 95 °C for 15 s, 55 °C for 40 s (1 min for HCO/LCO), and 72 °C for 40 s, plus a final elongation step of 72 °C for 3 min. To genotype a *Wolbachia* isolate, we amplified five MLST genes [31]. The amplicons (*COI* and MLST genes) were purified with exonuclease (ExoI) enzyme (New England Biolabs), and sequenced using a BrightDye Terminator Cycle Sequencing Kit (Nimagen). Full MLST profiles of four *Wolbachia* isolates were deposited in the PubMLST (https://pubmlst.org/wolbachia/) database, and all MLST alleles and fragments of the *COI* gene were deposited in the GenBank database (Table S1).

### 2.4. Quantitative Real-Time PCR Assay

We designed a qPCR assay to measure the relative number of *Wolbachia* genomes per beetle genome. A single copy gene per *Wolbachia* genome *fbpA*, and single copy gene per beetle genome *Inx2* were used to estimate the *Wolbachia* load in DNA samples. For a nuclear reference, the 750 bp region of the *Inx2* gene was amplified by nested PCR according to Che et al. [40], and then sequenced for confirmation of the target product (deposited in GenBank MT786531). Primers for qPCR were designed using the NCBI primer-BLAST tool (https://www.ncbi.nlm.nih.gov/tools/primer-blast/index.cgi?LINK_LOC=BlastHome). The primer sets Inx2prox (F 5′-AGTGGGTGTGCTTCGTATTGT-3′, R 5′-GTCCGCCTTACAGTCCTCG-3′), and fbpAprox (F 5′-CTGCGTGGCTGTTGGGTTTA-3′, R 5′-GCGCAGCATAGGCAATAACA-3′) amplified 143 bp and 188 bp fragments of *Inx2* and *fbpA* loci, respectively. Each qPCR mixture of 20 µL volume included 10 µL of ×2 qPCR HS-Taq mix (Biolabmix, Novosibirsk, Russia), 0.3 µM of each primer, and a 1 µL DNA sample that was in the range of 10–40 ng. All qPCRs were performed in duplicate on a CFX 96 Real-Time PCR Detection System (Bio-Rad Laboratories Inc., Hercules, CA, USA). Conditions of the qPCR were optimized by gradient PCR; we used 95 °C for 5 min, followed by 50 cycles of 95 °C for 10 s, and 63 °C for 30 s. Melt curves were generated to ensure specificity of the PCR product (65–95 °C with a step of 0.5 °C). Efficiencies of amplification and R^2^ were determined using a 5-fold dilution of a DNA sample for both loci (Inx2prox; E 98.21%, R^2^ 0.9987; fbpAprox: E 101.50%, R^2^ 0.9989).

### 2.5. Data Analysis

All statistical calculations were performed using Minitab 17.1.0 software (Minitab Inc., State College, PA, USA). The sequences were analyzed using FinchTV v. 1.4.0 (Geospiza Inc., Seattle, WA, USA). The alignments were generated using the MUSCLE algorithm [41]. For phylogenetic analysis of *P. proximus* mtDNA, the 620 bp region of the *COI* gene was used (38 sequences). To identify mtDNA haplogroups, we used the data from Kononov et al. [42]. Phylogenetic trees were reconstructed by the maximum likelihood algorithm in MEGA 6.0 [43], using the Tamura 3-parameter model for the *COI* gene and the GTR model for MLST data. For the MLST data analysis, we also used four *Wolbachia* isolates of Scolytinae species (Seqence Types (STs): 138, 207, 208, 210), seven STs (1, 8, 9, 35, 62, 19, 90) as *Wolbachia* supergroup references, and three STs (184, 303, 482) that had similar haplotype characteristics to ST-533 (Table S2; SM1). Each allele of ST-533 was independently analyzed for supergroup clustering to check the inter-supergroup recombination of *Wolbachia* haplotypes [8,9]. *Wolbachia* load was measured as a relative copy number (fbpA/Inx2) = 2 ^Cq(Inx2)−Cq(fbpA)^. Because the efficiencies of both primer sets were close to 100%, the amplification differences were not taken into account in calculations.

## 3. Results

### 3.1. Wolbachia Infection Rates and Wolbachia Titer

*Wolbachia* prevalence in populations of P. proximus was initially estimated by conventional PCR, followed by symbiont negative DNA samples being rechecked by nested PCR (Table S3). Finally, we estimated Wolbachia titer by real time quantitative PCR across local collections.

#### 3.1.1. Conventional PCR

The *Wolbachia* symbionts were found in 12 out of 13 *P. proximus* samples (Table 2). The only *Wolbachia*-free sample was Tomarinsky-2, Sakhalin, with a sample size of N = 8. The infection rates varied among sampling sites from 39% to 77% and were not statistically different between the studied regions (Pearson’s chi-square, *p* = 0.237).

Surprisingly, we found a great difference in *Wolbachia* prevalence for males and females, with 6 out of 13 samples being partially or totally subdivided based on sex. The proportion of infected females was significantly higher than males: across all collections *p* < 0.001; in localities, for three populations *p* < 0.001; for two populations *p* < 0.05; and for one *p* = 0.138.

#### 3.1.2. Nested PCR

Because a significant difference in *Wolbachia* rates within populations based on the sex of individuals was observed, we tried to reexamine all *Wolbachia* negative DNA samples by nested PCR. In total, we analyzed 189 samples, some of which were checked for two loci. As a result, 69 new infected samples were found. Previously seemingly *Wolbachia*-free samples from Tomarinsky-2 appeared to be nearly totally infected (7 out of 8). Taking into account the results based on both conventional and nested PCR, the infection rates varied from 48% to 92%. The infection rates in Udmurtia and Sakhalin were higher than in Tomsk and Krasnoyarsk (Fisher exact test for each of the four comparisons, *p* < 0.05 and *p* < 0.001).

Overall differences between infected females and males remained significant (Fisher exact test *p* < 0.01); however, in terms of localities, significant differences were only found in Tomarinsky-1 (*p* < 0.05) and Bakcharsky (*p* < 0.01) (Table 2).

#### 3.1.3. Estimation of Wolbachia Titer by qPCR

**Figure 2:**
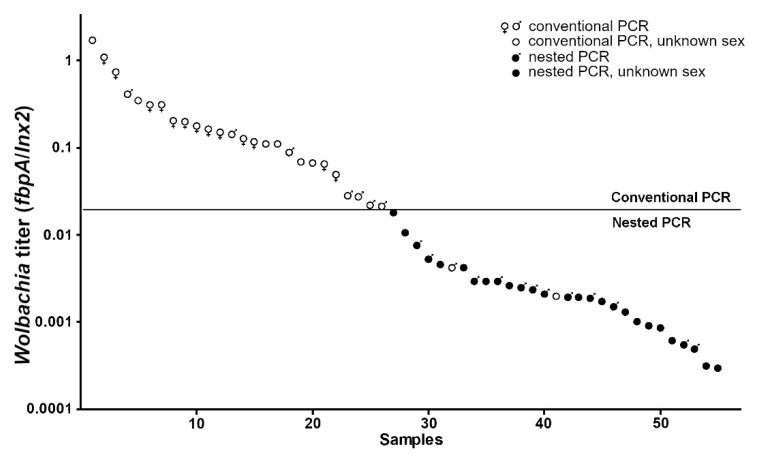
*Wolbachia* titer of *P. proximus* estimated by quantitative real time PCR.

The values of *Wolbachia* genomes per beetle genome were measured by real-time quantitative PCR. Here, we used 27 conventional-positive and 28 nested-positive samples across all regions; among them were 13 females, 20 males, and 22 samples with undetermined sex. Variation of the *Wolbachia* copy number (*fbpA*/*Inx2*) was in the range of 2.9 × 10^−4^ to 1.73. Our results for the conventional and nested PCR were in a good agreement with the estimation of *Wolbachia* load by qPCR. The threshold of the two PCR approaches was about 0.018–0.020 (Figure 2). The differences between the most heavily and lowest infected females was 22-fold, and the difference was 864-fold in males. The median of the *Wolbachia* titer was 0.1792 in females and 0.0035 in males, which was a significant difference (U Mann–Whitney *p* < 0.0001). In particular, there were no females below the threshold, whereas there were seven males (detected by conventional PCR) above it. In two cases, *Wolbachia* infection was successfully detected by conventional PCR, although the infection titers were ~0.002 and 0.005.

### 3.2. Characterization of Wolbachia Isolates and Mitochondrial DNA

**Figure 3:**
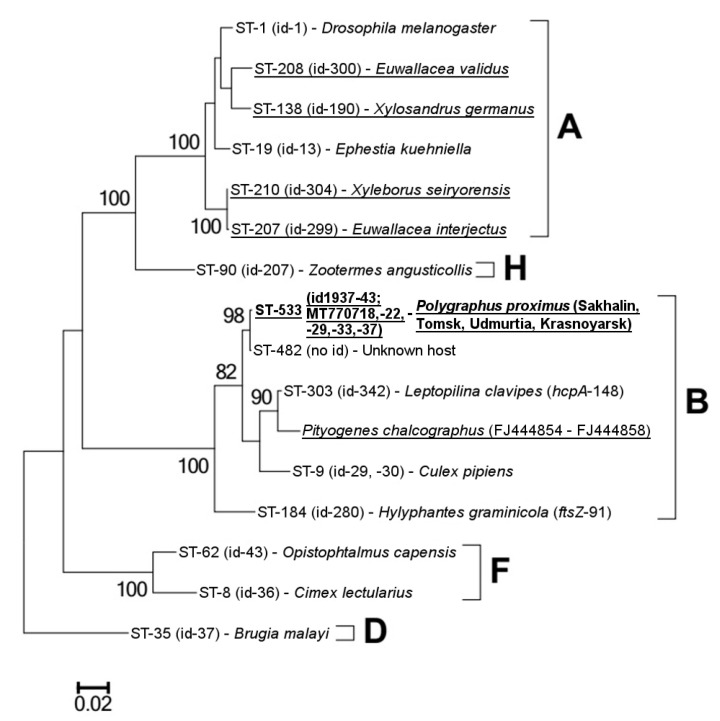
The maximum likelihood (ML) phylogenetic tree of *Wolbachia* isolates was reconstructed based on concatenated sequences of five multilocus sequence typing (MLST) genes using the GTR model of nucleotide replacement. The sequence type (ST), ID (id) numbers from the PubMLST database, and GenBank accession numbers, with host species, supergroups, and bootstrap values higher than 75 (1000 replicates), are indicated. *Wolbachia* isolates of the Scolytinae species are underlined. Studied isolates of ST-533 are **in bold**.

Complete MLST profiles were obtained for four *Wolbachia* isolates (SM-1) from the Tomsk, Krasnoyarsk and Sakhalin regions; all were identical and corresponded to the ST-533 haplotype. Alleles *ftsZ-263* and *fbpA-461* were first characterized in the PubMLST database; then, we checked them in the GenBank database using the BLASTn tool and confirmed their uniqueness. For this reason, we characterized additional isolates from populations of Udmurtia, Tomsk, and Krasnoyarsk (IDs 1940–1943; SM-1) with regard to the *ftsZ* and *fbpA* genes and did not detect any other alleles. The concatenated sequence of ST-533 and each MLST allele was clustered into supergroup B (Figure 3 and Table S2). The ST-482 haplotype was the closest to ST-533, and there were three substitutions in the *ftsZ* and *fbpA* loci. Unfortunately, the host of ST-482 was not indicated in the PubMLST database (an isolate was not registered). The *Wolbachia* haplotype of *Pityogenes chalcographus* (Scolytinae) was also clustered into supergroup B, with a total of 72 substitutions from ST-533. *Wolbachia* infection of *Leptopilina clavipes* (parasitoid wasp) had the same *hcpA-148* as ST-533 and ST-482; however, its haplotype (ST-303) was genetically closer to the isolate of *P. chalcographus* than that of *P. proximus.* The *ftsZ-91* allele of *Wolbachia* from *Hylyphantes graminicola* (spider) differed from *ftsZ*-263 of *w*Prox by only two substitutions, although other alleles were found to have much greater differences.

**Figure 4:**
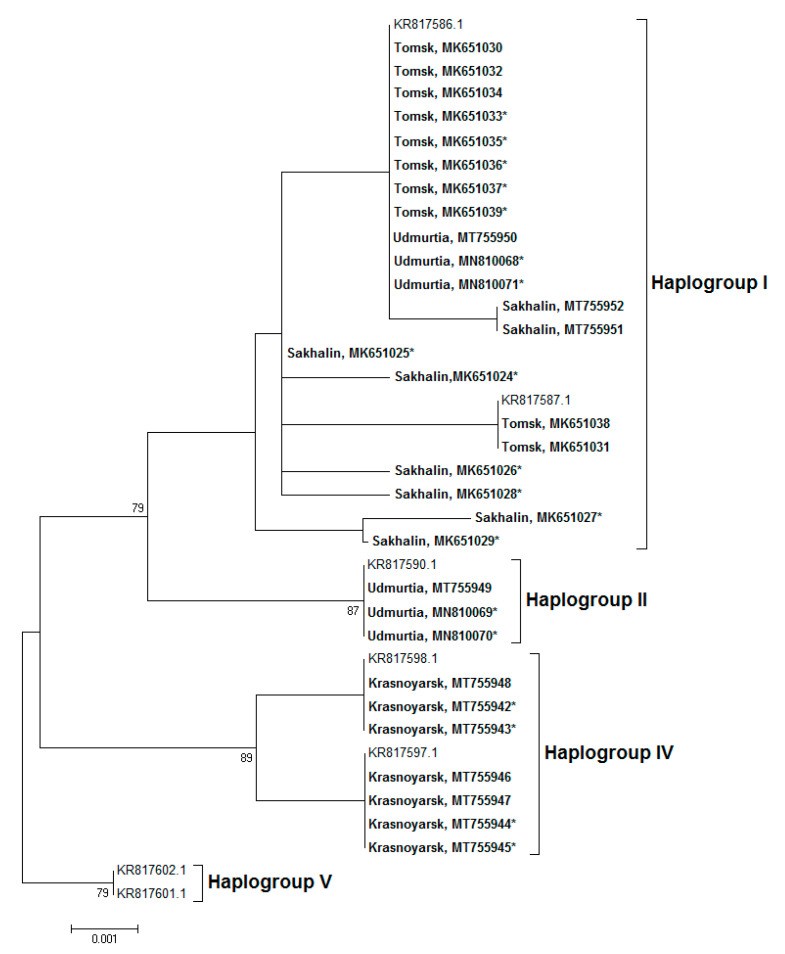
The ML phylogenetic tree of *P. proximus* was reconstructed with a Tamura 3-parameter model of nucleotide replacement based on sequences of a 620 bp region of the *COI* gene. Regions of collection, GenBank accession numbers, and bootstrap values higher than 75 (1000 replicates) are indicated. Samples of the study are indicated **in bold**. *Wolbachia*-infected samples are indicated with an asterisk (*). Haplogroups of mtDNA follow Kononov et al. [42].

As *Wolbachia* infection was uniform across a vast territory, we analyzed a 620 bp barcode region of the *COI* gene to characterize maternal inheritance in more detail. In particular, we aimed to determine the mtDNA haplotype of infected and uninfected *P. proximus* specimens at all sampling sites. As references, we used data from Kononov et al. [42], where five mtDNA haplogroups have been described. The analyzed mtDNA of infected and uninfected samples of the Sakhalin and Tomsk collections belonged to haplogroup I (Figure 4). In Udmurtia they belonged to haplogroups I and II, and in Krasnoyarsk haplogroup IV was detected. Thus, we did not find dependence between the *Wolbachia* infection status and mtDNA haplogroup.

## 4. Discussion

*Wolbachia* symbionts in the subfamily Scolytinae have previously been found in 11 species of 8 genera [26,27,28,29,30], and many of these species are characterized by high infection rates (70–100%). Here, we first provided data on *Wolbachia* infection in populations of *P. proximus* in four distant regions of its native and invasive ranges. The overall infection rate in the studied populations was 68% (95% confidence interval, 63–73%). Surprisingly, the infection rate of females was higher than in males (79.7% vs. 61.8%; Fisher exact test *p* = 0.003). Moreover, most of the infected males were characterized by significantly lower *Wolbachia* titers than the females. Genetic diversity of *Wolbachia* in *P. proximus* populations indicated only one strain in the vast studied territory, which is associated with at least three out of five mitochondrial haplogroups.

Maternal transmission of *Wolbachia* implies that the symbiont has no future in males; therefore, it seemed logical that *Wolbachia* could be absent in males. We have not found other reports of sex-specific *Wolbachia* distribution in other populations, as is the case with single infected *P. proximus*. However, Arai et al. [44] reported, in an experiment on subdividing triple infection, a 79% transmission rate for the *w*Hm-b strain in males of *Homona magnanima* (Lepidoptera: Tortricidae) versus a rate of 100% in daughters. Dutton and Sinkins [45], in double infected *Aedes albopictus*, found *w*AlbA loss in males. Further, Tortosa et al. [46] confirmed a loss of the *w*AlbA strain and demonstrated that wild females were double infected, whereas significant proportions of wild males were only single infected. There is a prediction of reduced expression of cytoplasmic incompatibility (CI) in males through selection of the host genome [47], and *Wolbachia* loss in males could be considered [46]. Therefore, if *Wolbachia* maintenance in a host population is based on CI, CI repression due to *Wolbachia* loss in males should lead to a loss of infection in a whole population. We do not have sufficient information to infer the effect(s) of *Wolbachia* on *P. proximus* biology, although the *Wolbachia*-inducing phenomena that lead to a sex ratio bias should be excluded, because we observed the primary sex ratio to be 1:1. There is a question as to whether *Wolbachia* in *P. proximus* is driven by CI and therefore will be lost in future, or high *Wolbachia* rates maintained by mutualistic effects for females, as, for instance, has been shown for *Wolbachia*-infected females of the coffee berry borer *Hypothenemus hampei* (Scolytinae), which produce more eggs than tetracycline-treated females [12].

The uninfected *P. proximus* males are likely the result of *Wolbachia* loss in ontogenesis. Most infected males are characterized by low *Wolbachia* density over all of the studied regions. Similar results were found for the *w*AlbA strain of *Ae. albopictus* [45,46]. In Scolytine, cases of low *Wolbachia* titers were found in populations of *Pityogenes chalcographus* [29]; however, there was no comparison between males and females. Therefore, there are few examples of sex-specific differences in *Wolbachia* titers, whereas there are a number of low symbiont titers in other species [29,48,49,50,51,52,53,54,55].

## Figures and Tables

**Table 1:** Collection of *Polygraphus proximus*.

Locality No.	Sampling Sites, Coordinates (Where Available), and Date of Collection	N	Sex		
			Unknown	♀	♂
**Sakhalin province (in total)**		**60**	**23**	**24**	**13**
**1**	Tomarinsky-1, 48°29′22.2″ N 142°01′49.7″ E, 8 June 2018	37	-	24	13
**2**	Tomarinsky-2, 48°30′26.6″ N 142°00′43.9″ E, 8 June 2018	8	8	-	-
**3**	Yuzhno-Sakhalinsk, 46°57′50.8″ N 142°45′16.7″ E, 11 June 2018	15	15	-	-
**Krasnoyarsk krai (in total)**		**48**	-	**34**	**14**
**4**	Bolshemurtinsky district, 25 December 2019	2	-	1	1
**5**	Emelianovsk forestry, December 2019	16	-	8	8
**6**	Kozulsk forestry, 12–17 February 2020	30	-	25	5
**Tomsk province (in total)**		**271**	**148**	**63**	**60**
**7**	Tomsky, 56°27′55.0″ N 85°06′46.0″ E, 28 August 2018	33	33	-	-
**8**	Krivosheinsky, 57°24′27.0″ N 83°55′16.0″ E, 7 August 2018	26	26	-	-
**9**	Molchanovsky, 57°29′34.0″ N 84°16′27.0″ E, 18 August 2018	31	31	-	-
**10**	Verkhneketsky, 58°23′30.2″ N 84°06′35.8″ E, 29 August 2019	89	-	46	43
**11**	Chainsky, 57°47′17.0″ N 82°12′34.0″ E, 10 August 2018	34	34	-	-
**12**	Bakcharsky, 57°16′35.0″ N 81°30′18.0″ E, 15 August 2018	58	24	17	17
	**Udmurtia**				
**13**	Malopurginsky district, 56°38′40.0″ N 53°05′57.6″ E, 11 October 2019	38	5	17	16
**Total:**		**417**	**176**	**138**	**103**

**Table 2:** *Wolbachia* infection in *P. proximus*.

Locality No.	Region, Locality	Conventional PCR		Conventional and Nested PCR	
		N_un_^inf^/N♀^inf^/N♂^inf^	Total %^inf^ (95%CI)	N_un_^inf^/N♀^inf^/N♂^inf^	Total %^inf^ (95%CI)
	**Sakhalin province (in total)**	**8/23/4**	**58.33 (44.88–70.93)**	**20/23/9**	**86.67 (75.41–94.06)**
**1**	Tomarinsky-1	0/23/4	72.97 (55.88–86.21)	0/23/9	86.49 (71.23–95.46)
**2**	Tomarinsky-2	0/0/0	-	7/0/0	87.5 (47.35–99.68)
**3**	Yuzhno-Sakhalinsk	8/0/0	53.33 (26.59–78.73)	13/0/0	86.67 (59.54–98.34)
	**Krasnoyarsk Krai (in total)**	**0/25/4**	**60.42 (45.27–74.23)**	**0/25/6**	**64.58 (49.46–77.84)**
**4**	Bolshemurtinsky district	0/1/0	-	0/1/0	-
**5**	Emelianovsk forestry	0/8/3	68.75 (41.34–88.98)	0/8/5	81.25 (54.35–95.95)
**6**	Kozulsk forestry	0/16/1	56.67 (37.43–74.54)	0/16/1	56.67 (37.43–74.54)
	**Tomsk province (in total)**	**74/41/15**	**47.97 (41.89–54.1)**	**87/45/34**	**61.26 (55.17–67.09)**
**7**	Tomsky	20/0/0	60.61 (42.14–77.09)	22/0/0	66.67 (48.17–82.04)
**8**	Krivosheinsky	20/0/0	76.92 (56.35–91.03)	21/0/0	80.77 (60.65–93.45)
**9**	Molchanovsky	12/0/0	38.71 (21.85–57.81)	15/0/0	48.39 (30.15–66.94)
**10**	Verkhneketsky	1/24/12	41.57 (31.21–52.51)	1/28/24	59.55 (48.62–69.83)
**11**	Chainsky	15/0/0	44.12 (27.19–62.11)	19/0/0	55.88 (37.89–72.82)
**12**	Bakcharsky	6/17/3	44.83 (31.74–58.46)	9/17/10	62.07 (48.37–74.49)
	**Udmurtia**				
**13**	Malopurginsky district	3/17/1	55.26 (38.30–71.38)	4/17/14	92.11 (78.62–98.34)
**Total:**		**85/106/24**	**51.56 (46.65–56.45)**	**111/110/63**	**68.11 (63.40–72.56)**

**N_un_^inf^**—*Wolbachia*-infected samples with undetermined sex; **N♀^inf^**—*Wolbachia*-infected females; **N♂^inf^**—*Wolbachia*-infected males.

## Supplementary Materials

The following are available online at https://www.mdpi.com/2075-4450/11/8/547/s1. Table S1. GenBank accession numbers of *Wolbachia* MLST genes and mtDNA *COI* gene used for phylogenetic analysis. Table S2. MLST profiles of *Wolbachia* isolates. Table S3. Data of conventional, nested PCR, and *Wolbachia* titer. Supplementary Material 1. Fasta file of concatenated sequences of five *Wolbachia* MLST genes used in this study.
